# Environmental Geochemical Analysis in the Yanomami Indigenous Land, Mucajaí River Basin, State of Roraima, Brazil

**DOI:** 10.3390/toxics11100861

**Published:** 2023-10-14

**Authors:** Patricia Duringer Jacques, Eduardo Paim Viglio, Daniel de Oliveira d’El Rei Pinto

**Affiliations:** 1 CPRM—Geological Survey of Brazil, Rio de Janeiro 22.290-255, Brazil; eduardo.viglio@sgb.gov.br; 2 Habitat Geo and Fundação Oswaldo Cruz, Rio de Janeiro 22.290-255, Brazil; daniel@habitatgeo.com.br

**Keywords:** environmental geochemical, Yanomami Indigenous Land, mercury contamination

## Abstract

The Yanomami Indigenous Land in the Amazon has a long history of illegal artisanal gold mining, leading to concerns about mercury (Hg) contamination. This study has conducted a geochemical analysis to assess Hg contamination from these mining activities. Geological materials, including river water and stream sediments, were collected from 14 predetermined points based on the Geological Survey of Brazil’s methodology. The results revealed that water samples did not show Hg contamination above the limits set by the National Council of the Environment (Conama) Resolution 357. However, two stream sediment samples, particularly PJS009 and PJS010 collected from the Mucajaí River, exceeded the Conama Resolution 454’s limit of 0.17 mg/kg. A Hg content of 0.344 mg/kg was found in the sediment sample PJS009, the one collected further upstream in the Mucajaí River, and 1.386 mg/kg was found in sample PJS010, also in the Mucajaí River in the region shortly before the Fumaça Waterfall, indicating that the sediments of the Mucajaí River may be contaminated with Hg from the Fumaça Waterfall upstream.

## 1. Introduction

There is a long history of metal mining in Brazil, which has polluted geological materials such as sediments, waters, oceans, and soils. These geological materials can reflect anthropogenic contamination sources [1,2,3,4] from the Anthropocene Epoch [5,6], when the human race began altering the planet, including long-term global geologic processes, at an increasing rate. This is even the case in places that should be isolated, such as regions designated as indigenous land in the Amazon region.

Being the region with the greatest biodiversity on the planet, the Amazon Region is home to more than half of the indigenous peoples of Brazil. They have developed a traditional way of life, intrinsically marked by their relationship with the environment, provided by the abundance of natural resources.

Although there are marks in the landscape from settlements, structures for managing water and fishing, and small fires from the beginning of the Christian era, it was the contact with European civilization from the 16th century onwards that caused the man–nature relationship in the Amazon to change.

New pressures on the biome are related, among other factors, to population growth, the search for natural resources, the rise of the capitalism model, technological development, the increase in land value, and the expansion of the agricultural frontier [7,8]. Intense migratory flows created cities and municipalities and boosted old urban centers with the discovery of gold deposits in the region. Along with other sociospatial fronts and processes, mining contributed to occupying the demographic frontier and consolidating the regional space [9].

These pressures have been growing over the years. According to data from the MapBiomas Project [10], from 1985 to 2021, there was a loss of 44.16 million hectares of forest cover in the Amazon biome. On the other hand, in the same period, there was an increase of 44.52 million hectares of areas occupied by agricultural activities, 225,867 ha of urban areas, and 217,387 ha of areas occupied by mining in the Amazon.

Widespread in the region, mining has been developed in different territories and, unfortunately, indigenous lands did not escape this process. Artisanal mining (garimpo) in indigenous lands, in addition to being illegal, is marked by the indiscriminate use of mercury—a metal used to separate gold from sediments, causing serious impacts on the environment and human health. After its release into the environment, this metal undergoes several chemical transformations, being incorporated into the food chain and, thus, reaching humans and being able to cause, in addition to sensory and motor neurological problems, other serious illnesses. The presence of mercury in the food chain is especially harmful in the Amazon since fishing is an essential activity [11]. There is a wide range of exposure, with average levels of mercury in hair samples above 15 µg/g in several Amazonian communities, placing them among the highest levels reported in the world [12,13].

One of the most affected territories by illegal gold mining in the Amazon is the Yanomami Indigenous Land, a place that has seen an explosion in prospecting activity in recent years, based on data from the Mapbiomas project [10]. This data highlights that the area dedicated to this activity surpassed 650 hectares in 2019, reaching 920 and 1,556 hectares, respectively, in subsequent years. Despite the advances obtained with remote sensing methodologies for environmental monitoring in Brazil, some regional specificities are probably not detected in this analysis, as is the case for mining carried out on rafts routinely seen in several Amazonian water bodies. They are smaller than the spatial resolution of Landsat images (30 m) and also migrate easily via the great rivers of the region. This fact was corroborated during this field work when an illegal mining raft was observed to be active on the Mucajaí River (latitude 2.759953°; longitude -62.290173°—Geographical Coordinates/SIRGAS Horizontal Datum 2000), close to the Fumaça Waterfall. However, it was “imperceptible” in Landsat images and in many other images with spatial resolution.

Since 2016, there has been some accelerated growth according to data from the National Institute of Spatial Research (INPE) [13], including in the Yanomami Indigenous Lands, caused by illegal mining, which increased from 0.1 km^2^ in 2016 to 4.2 km^2^ in 2020. There was an interruption in the identification of artisanal mining between the years 1998 and 2015. This may be related to the limitations of identifying mining by remote management or even the fact that mining in the Yanomami Indigenous Land (IL) in the 1980s and 1990s took place in isolated spots in the Serra do Surucucu. Moreover, tracks were bombed by the federal government, and mining was extinguished in the area. The return in 2017 was due to newly discovered gold and tantalite in another region.

Overlapping data from the National Mining Agency (ANM) within the limits of the Yanomami territory and its immediate surroundings of 5 km reveal the existence of 624 mining processes in different phases, the majority of which (94%) are in the research requirements phase, which is the first step to start the whole mineral process. 

According to research carried out in the geographic information system of the National Mining Agency (SIGMINE) [14], the most coveted substance is gold, with more than 700,000 hectares required in that spatial area for this metal of great market value. Despite gold being the main target of the mining sector in the region, cassiterite and tantalite also arouse great interest with a required area of over 320,000 hectares.

A study published by the Geological Survey of Brazil (SGB) [15] in 2017 produced the geochemical atlas of the state of Roraima for approximately 54 chemical elements based on the collection stations of river water, river sediments, and soils from 2011. Among them, 429 river sediment samples were collected throughout the state, with the exception of indigenous areas in the state (including the Mucajaí River Basin). In the case of sediment samples for Hg analysis, only 204 points out of the 429 collected (48%) had an Hg value above the detection limit. The minimum value detected was 0.005 mg/kg and the maximum value was 1.05 mg/kg, the latter recorded in the Jufari River Basin. In the geochemical atlas of the state of Roraima, around 10% of stream sediment samples and 30% of soil samples showed content above the crustal average for Hg (0.08 mg/kg). Unfortunately, there was no collection of samples in the area studied in this article.

Bearing in mind the impact of such prospecting activity on the health of the Yanomami indigenous people who live on the banks of the Mucajaí River, with a focus on exposure to mercury, this paper analyzes the geological material (river water and stream sediment) collected in the Hydrographic Basin of the Mucajaí River (Roraima, Brazil), in October 2022, using the methodology developed by the Geological Survey of Brazil for a low-density geochemical survey.

This work is part of a research project of the Oswaldo Cruz Foundation—FIOCRUZ, known as “Impact of mercury in protected areas and forest peoples in the Eastern Amazon: an integrated health-environment approach”, which obtained the authorization of Entry into Indigenous Land number 67/AAEP/PRES/2022 from the National Indian foundation—FUNAI on behalf of the coordinator of the Project, Dr. Paulo Cesar Basta, allowing 23 researchers to conduct multidisciplinary research.

## 2. Materials and Methods

### 2.1. Study Area and Sampling Points 

**Figure 1:**
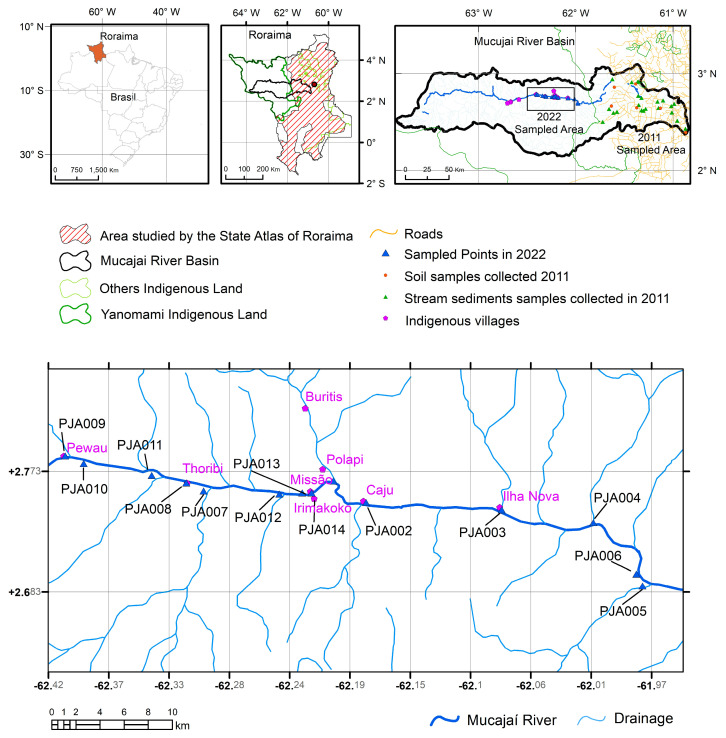
Area of studies and points of collection of a sample of geological material carried out (coordinate system Datum WGS 1984).

The study area is located in the State of Roraima, Brazil, on the upper course of the Mucajaí River, where 14 sample points of geological material from the rivers (14 samples of river water and 14 samples of stream sediment from the banks) were collected, which were sent for laboratory analysis (Figure 1). Sampling points are described in Table 1.

### 2.2. Data Collection

Field materials and equipment used for the collection were as follows: (i) Tablet with an application for recording the sample data in the SGB/CPRM geochemistry database (point coordinates, physical–chemical parameters obtained by the probe and records made in loco by the researcher); (ii) probe (AquaRead multiparameter meter) for recording pH, temperature, dissolved oxygen, electrical conductivity, Eh, and turbidity; (iii) bucket, plastic beakers, disposable syringes without tips, 0.45 µm millipore filters, sterilized polyethylene tubes of 50 mL, 20 mL of nitric acid, thermal box to keep the samples under refrigeration after collection; (iv) striped plastic bags, insulating tape, and permanent marker pens for sample identification and storage; (v) water samples were placed in thermal boxes at an average temperature of 10 °C.

**Figure 2:**
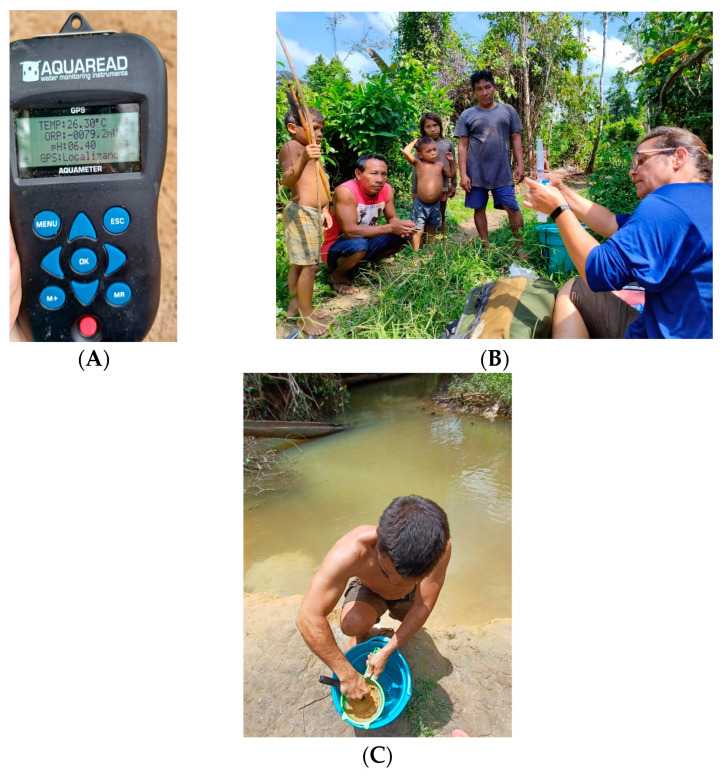
(**A**) Probe with some water measurements. (**B**) Water collection. (**C**) Stream sediment collection with sieving.

The sampling points were previously loaded on the tablet and on the GPS device (Garmin GPSmap 62sc model) and navigation was promoted along the Mucajaí River to the tributaries that were sampled. Upon reaching the previously programmed point, the multiparametric meter was turned on and the probe was placed inside the bucket with a water sample where data on temperature, turbidity, pH, dissolved oxygen, electrical conductivity, and salinity were measured (Figure 2A). 

Water was collected using a syringe, removing water from the beaker, and attaching the filter to its tip. The water was then inserted into the previously identified polyethylene tube and the first 50 mL (anions) were filtered. (Figure 2B).

The process was repeated in a second 50 mL tube acidified with 10 drops of nitric acid and identified with red ribbons for cation analysis. A third collection was performed without a filter but with acidification of the samples (10 drops of nitric acid) for mercury analysis. They were identified with the name of the sample and the sampled river and kept in the thermal boxes.

The stream sediments collection was carried out on the chosen margins with a predominance of fine sediments. The finer sediments were collected and placed directly in the plastic bag when composed of clay. In the case of sandier sediments, these were sieved through a 20# sieve. Only the material that passed this sieving was collected. Each sample contains about 1 kg of fine material (Figure 2C).

The samples were recorded on the tablet, with numerous characteristics noted, such as the following: width of the river; depth; flow speed; water level; type of vegetation on the banks; water color; sediment color; sediment composition; collection margin; all physical-chemical records measured; coordinates and elevation obtained with GPS. 

### 2.3. Laboratories

After collection, the water samples were sent to the Geological Survey of Brazil-CPRM laboratory, located in Manaus, Brazil (LAMIN—MA), and analyzed via ICP-OES (Atomic Emission Spectrometry with Plasma Source) for 27 cations (Al, As, B, Be, Ba, Ca, Co, Cd, Cu, Cr, Li, Fe, Hg, K, Mg, Mn, Mo, Na, Ni, Pb, Se, Si, Sb, Sn, Sr, Ti, V, and Zn) and via ionic chromatography for 7 anions (fluoride, chloride, bromide, nitrite, nitrate, sulfate, and phosphate). Atomic absorption analyses for total Hg (DMA-80) were also performed.

The stream sediment samples were previously dried in ovens at a low temperature (50 °C), homogenized, and sieved at 80#, the passer being crushed at 150#, and acqua regia was used for an acid attack of the sediment. Analyses were performed via ICP-OES or ICP-MS (Inductively Coupled Plasma Mass Spectrometry) for 53 elements (Ag, Al, As, Au, B, Ba, Be, Bi, Ca, Cd, Ce, Co, Cr, Cs, Cu, Fe, Ga, Ge, Hf, Hg, In, K, La, Li, Mg, Mn, Mo, Na, Ni, P, Pb, Pd, Pt, Rb, Re, S, Sb, Sc, Se, Sn, Sr, Ta, Te, Th, Ti, Tl, U, V, W, Y, Zn, and Zr).

### 2.4. Legal References Used

In Brazil, Conama (National Council of the Environment) is the official agency responsible for determining the limits and quality standards for water, soil, sediment, and effluents, defining the quality values for each.

The maximum permissible value for freshwater class I of Conama Resolution 357 of 17 March 2005 [16] or the groundwater parameters of Conama Resolution 396 of 2008 [17] was applied for the evaluation of water quality. The maximum value allowed by the Ministry of Health Ordinance No. 2914 of 2011 [18] can also be used. In the absence of indications, the prevention values (Threshold Effects Level—TEL) from the 2008 NOAA Screening Quick Reference Tables [19] or the Guidelines for drinking-water quality from the World Health Organization, WHO, from 2011 [20] were applied to the assessment of sediments’ quality.

For the bottom sediment samples, level 1 values for freshwater from Conama resolution 454 of 11 January 2012 [21] were used for dredged sediments or the TEL of NOAA—SQuiRT for inorganic solids in February 2008 [20].

## 3. Results

All water test results for Hg came out negative. However, in four samples, the values exceeded the limits set by Conama Resolution 357 for Fe (0.300 mg/L), and in one sample, they exceeded the limit for Al (0.100 mg/L). In terms of the physico–chemical parameters, one sample showed a conductivity value above the recommended limit of 100 µS/cm, and two samples exhibited extremely high turbidity values exceeding 1,000 NTU. The following elements—As, B, Be, Cd, Co, Cr, Cu, Hg, Li, Mo, Ni, Pb, Sb, Se, Sn, Ti, and V—were not detected in the water samples. On the other hand, the levels of elements Ba, Ca, K, Mg, Mn, Na, Si, Sr, and Zn either remained below the limits specified in the current resolutions or were very low.

The results can be seen in Table 2 and their statistical summary in Table 3.

Two sediment samples showed mercury levels above level 1 of Conama Resolution 454. Hg contents of 0.344 mg/kg were found in the sediment sample PJS009, the one collected further upstream in the Mucajaí River, and 1.386 mg/kg in sample PJS010, which was also collected in the Mucajaí River in the region shortly before the Fumaça Waterfall, indicating that the sediments of the Mucajaí River may be contaminated with Hg from Fumaça Waterfall upstream. The presence of Hg in lower concentrations was found in the samples from the Jacaré and Guximaí rivers (water sample numbers PJA005 and PJA007, respectively), indicating the presence of possible gold mines towards their headwaters. High values were found for Al (water sample PJA008) and Fe (water samples PJ004, PJA005, PJA006, and PJA007), and low values for As, Au, Ba, Be, Ca, Cd, Ce, Co, Cr, Cs, Cu, Ga, Ge, In, K, La, Li, Mg, Mn, Mo, Na, Ni, P, Pb, Rb, Sc, Sn, Sr, Th, Ti, U, V, W, Y, and Zn. The elements Ag, B, Bi, Hf, Nb, Pd, Pt, Re, S, Sb, Se, Ta, Te, Tl, and Zr were not detected.

The results can be seen in their statistical summary in Table 4 and in the results in Table 5.

**Figure 3:**
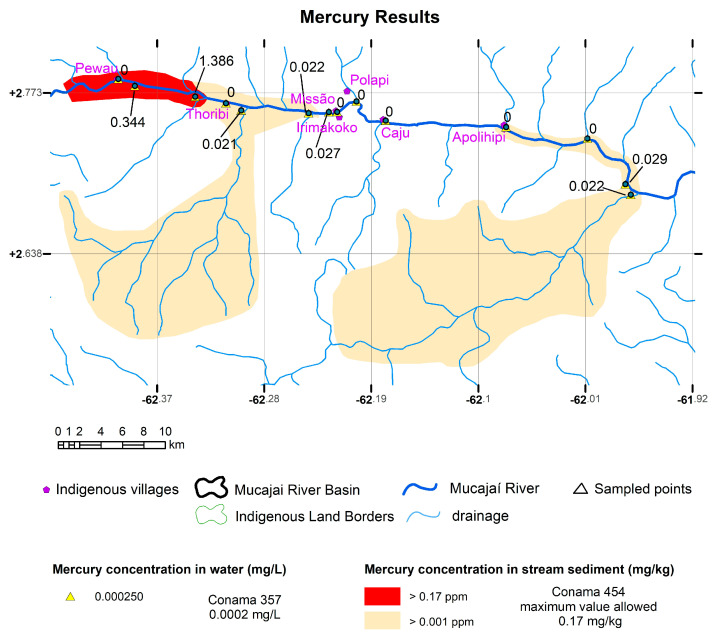
Map of mercury concentration in sediment (mg/kg) with the points collected from water in mg/L. Mercury was not detected in water, only in sediments (using the Conama 454 limits <0.0002 mg/L for water and 0.17 mg/kg for Hg). Coordinate system—Datum WGS 1984).

**Figure 4:**
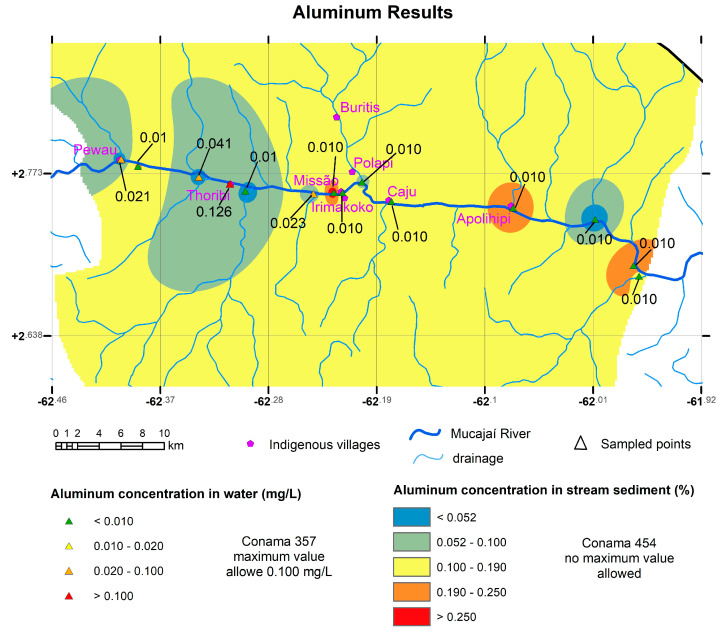
Map of aluminum concentration in sediment (mg/kg) with the points collected from water in mg/L. Both the water samples and the sediments have high values (using the Conama 357 limit of 0.100 mg/L for water). Coordinate system Datum WGS 1984.

**Figure 5:**
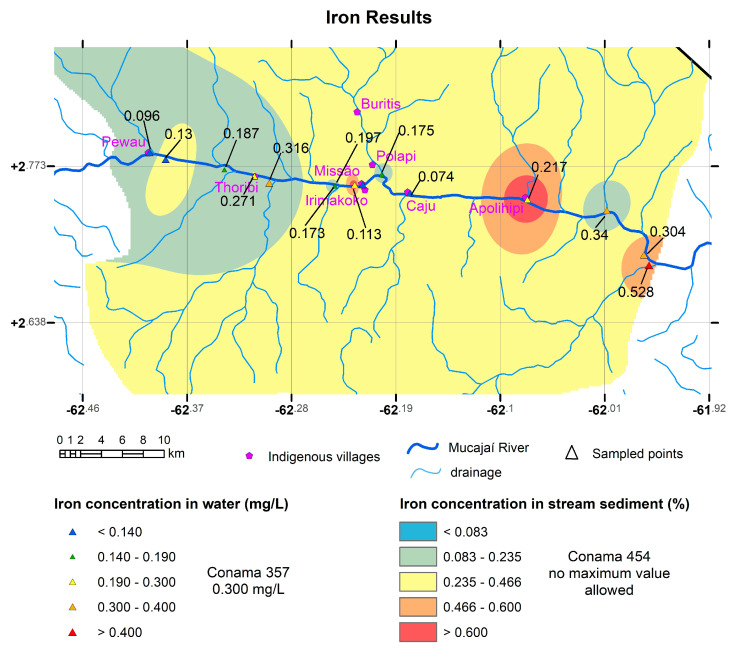
Map of iron concentration in sediment (mg/kg) with the points collected from water in mg/L. Both the water samples and the sediments have high values (using the Conama 357 limit of 0.300 mg/L for water). Coordinate system Datum WGS 1984.

Maps were generated for the elements Hg, Al, and Fe in sediment, which are shown in Figure 3, Figure 4, and Figure 5, respectively. In the region of influence of the sampled basins, a polygon was delimited within which a raster surface was generated that represents the probable spatial and mathematical variation in the concentrations of elements in the stream sediments. For Hg, the sampled basins were delimited, indicating those with the presence of Hg and the region of the Mucajaí River from Fumaça Waterfall upstream were still open (because we were prevented from collecting samples further upstream for safety reasons), with values above the legislation.

For Al (Figure 4) and Fe (Figure 5), maps interpolated in the ArcGIS 10.8.2 software using the IDW (Inverse Distance Weighted) method are presented. 

## 4. Discussion

The high values of Al and Fe, in this work, are interpreted as a result of lateritization processes in the Amazon Region. Eroded materials from the rocks are carried to the river sediments. The entire Amazon region is affected by processes that concentrate metallic elements, mainly Fe, Al and Mn, in the horizons that develop lateritization processes, generating ferruginous, manganese and bauxite crusts. The physical weathering of these horizons carries these metals to the river, where they are deposited with the heaviest sediments and end up partly solubilized and incorporated into the water. It is normal for values above those defined by the Brazilian legislation to occur in Amazonian waters for Al and Fe.

The comparison of the distribution patterns and grades of Hg obtained by the SGB’s study Geochemical Atlas of the State of Roraima and the ones obtained by this present study show a similarity among the number of samples below the detectable level (about 50%) and also with 14% (two of the 14 samples) with levels above the crustal average.

The origin of Hg in the region deserves more detailed investigations to try to define the origin of the enrichment of Hg in these sediments. Mercury is a transition metal, dense, highly volatile, which rarely occurs free in nature and is liquid under room temperature conditions and the known ore mineral is cinnabar (HgS), whose main deposits are found in Spain [22]. There are no cinnabar mines in Brazil and in the Amazon region there is no occurrence of Hg. Therefore, the possibility of the origin of high levels of Hg in the sediments of the state of Roraima and the area of the watershed of the Mucajaí River is that it is anthropic contamination, arising from the activity of illegal mining, which uses Hg to amalgamate gold in prospecting and mining.

Part of the metallic mercury may be converted to methylmercury via the action of microorganisms that live in river sediment [23]. Studies carried out in 2021 by Crespo-López et al. [24] demonstrate that there are methanogenic bacteria in two large dams in the Amazon (Tucurí and Balbina), which promote the methylation of Hg. At the bottom of the lakes of these dams, there are favorable conditions for the existence of these bacteria, which allow the entry of MeHg into the food chain. According to the same study, methanogenic bacteria can transform Hg into MeHg both from water and directly from sediment. There are contaminated people and contaminated fish around the dams, downstream, and in regions quite far from the artisanal mining region.

In the Mucajaí River Basin, methylmercury exists and is incorporated by aquatic biota with higher concentrations for organisms at the top of the food chain. Many of these organisms are part of the traditional diet of the Amazonian population, especially in indigenous communities, where access to other food resources is reduced.

Several studies in the region point to the presence of methylmercury at levels higher than those recommended by FAO/WHO [25]. As an example of this scenario, a study conducted by de Vasconcelos et al. 2021 [26] reveals a prevalence of methylmercury contamination (MeHg ≥ 0.5 µg/g) of 53% of fish analyzed in the lower Rio Mucajaí.

Anthropogenic mercury contamination, especially in the Amazon region, poses a great risk to human health. The consumption of contaminated fish can cause various health effects. Specifically in the Amazon, cognitive skills loss, psychomotor alterations, and mental development problems were observed in children exposed during the first months of the prenatal period [27]. In adults, symptoms such as depression, aggressiveness, insomnia, motor coordination problems, and visual capacity are commonly reported due to chronic exposure to mercury [27,28].

## 5. Conclusions

The results obtained show the existence of mercury in the bottom sediment of part of the Mucajaí, Jacaré, and Guximaí rivers. The high values above those allowed by Conama 454 are found close to Fumaça Waterfall, and also the indigenous community of Pewau, probably coming from the mines in Alto Mucajaí, according to data from MapBiomass [10]. Hg beads are heavy and are usually transported to the bottom of the main channel. The sudden break in the water flow immediately above Fumaça Waterfall must be the cause of the high concentration found just before it.

The absence of Hg in the surface water sampled indicates that there are no active processes in the region that promote the solubilization of Hg, allowing the passage of Hg found in the sediment to the water. If methylated Hg is present in the food chain, it is necessary to carry out a specific study to describe and understand the processes operating at the sediment–water interface at the bottom of the river that promote this methylation. But it is possible that the region of quiet waters caused by the Fumaça Waterfall allows the existence of bacteria that cause the methylation of Hg directly from the sediment, allowing its entry into the food chain.

The next stage of the study should include the integration of the geological material with the biological material collected in the study area for the evaluation of human contamination via the contamination of fish and hair samples from local communities, especially the communities close to points PJS0010 and PJS0011, Pewau and Thoribi indigenous communities.

Based on the absence of cinnabar (ore mineral of Hg) and the fact that in the field, during the sample collection in the field, illegal mining dredgers were found in the Mucajaí River, it can be deduced that the contamination of the river sediment samples is of anthropic origin caused by illegal mining, as a consequence of the use of amalgams for gold extraction. 

## Figures and Tables

**Table 1:** Geographical coordinates, elevation, and sampling date of sampling locations.

Sample ID	Lat (N)	Long (W)	Date	Elevation	Location of Sampling Points
PJA001	2.76584	−62.20544	10-October-2022	185	Left bank of Klokonai River, near the left bank of Mucajaí River
PJA002	2.74977	−62.18100	10-October-2022	186	Left bank of Klaitabiu River
PJA003	2.74438	−62.07983	10-October-2022	182	Left bank of Baixukuau River
PJA004	2.73472	−62.01156	10-October-2022	184	Left bank of Sikaimabiu River
PJA005	2.68764	−61.97510	10-October-2022	171	Right bank of Jacaré River
PJA006	2.69650	−61.97961	10-October-2022	176	Right bank of the Mucajaí River. Sandbank abandoned by prospectors, with many pebbles.
PJA007	2.75834	−62.30223	11-October-2022	203	Right bank of Guximai River, a tributary of the right bank of the Mucajaí River
PJA008	2.76437	−62.31511	11-October-2022	209	Left bank of the Yalakapu Creek, a tributary of the left bank of the Mucajaí River
PJA009	2.78466	−62.40548	11-October-2022	221	Left bank of the Pewau River. Indigenous Village of Pewau
PJA010	2.77879	−62.39161	11-October-2022	212	Upper Mucajaí River
PJA011	2.76993	−62.34092	11-October-2022	211	Right bank of the Mucajaí River, near Fumaça Waterfall.
PJA012	2.75605	−62.24571	11-October-2022	189	Left bank of the Mucajaí River
PJA013	2.75697	−62.22869	12-October-2022	200	Left bank of the Maxthak-u River, a tributary of the left bank of the Mucajaí River.
PJA014	2.75700	−62.22169	12-October-2022	200	Left bank of the Mucajaí River. Iasasi Indigenous Village

**Table 2:** Results obtained in water samples.

Physico–Chemical Parameters							
Sample ID	Conductivity (µS/cm)	Temp (°C)	pH	Turbidity (NTU)	Eh (mV)	TDS (mg/L)	-
PJA001	57	24.90	6.44	23.30	−3.50	37	
PJA002	65	24.70	6.21	8.30	47.40	40	
PJA003	56	26.20	6.22	16.40	−0.50	36	
PJA004	82	-------	6.05	15.10	57.50	52	
PJA005	77	27.30	6.80	30.40	−119.70	49	
PJA006	47	27.50	7.06	1176.00	−149.70	31	
PJA007	118	24.98	6.78	2016.00	−16.10	78	
PJA008	70	24.00	6.41	33.40	−6.00	44	
PJA009	46	24.50	6.44	16.70	8.60	29	
PJA010	53	26.60	6.73	195.00	15.50	32	
PJA011	49	26.30	6.46	136.00	−87.10	31	
PJA012	48	26.70	6.35	176.00	−47.60	27	
PJA013	63	25.00	6.53	6.50	21.50	42	
PJA014	47	26.80	6.06	209.00	58.70	31	
**Anions**							
**Sample ID**	Bromide mg/L	Chloride mg/L	Fluoride mg/L	Phosphate mg/L	Nitrate mg/L	Nitrite mg/L	Sulfate mg/L
PJA001	0.005	3.9274	0.0427	0.005	0.4702	0.005	0.0782
PJA002	0.005	0.6541	0.0674	0.005	0.4639	0.005	0.005
PJA003	0.005	0.6885	0.0586	0.005	0.4903	0.005	0.0882
PJA004	0.005	0.7983	0.0573	0.005	0.5132	0.005	0.0653
PJA005	0.005	0.7815	0.0774	0.005	0.645	0.005	0.1737
PJA006	0.005	0.5686	0.0439	0.005	0.8243	0.005	0.2050
PJA007	0.005	0.6466	0.0396	0.005	0.6286	0.005	0.0911
PJA008	0.01505	0.7984	0.0426	0.005	1.0462	0.0101	0.2494
PJA009	0.005	0.5171	0.0355	0.005	0.4726	0.005	0.0434
PJA010	0.005	0.4845	0.0457	0.005	0.728	0.005	0.2221
PJA011	0.005	0.5298	0.0399	0.005	0.7090	0.005	0.2057
PJA012	0.005	0.5061	0.0434	0.005	0.8378	0.005	0.4135
PJA013	0.005	0.7459	0.0778	0.01275	0.3616	0.005	0.1958
PJA014	0.005	0.7904	0.0444	0.005	0.8783	0.005	0.2568
**Cations**							
**Sample ID**	Al mg/L	Ba mg/L	Ca mg/L	Fe mg/L	Hg mg/L	K mg/L	
PJA001	0.01	0.03	1.507	0.175	0.00025	1.696	
PJA002	0.01	0.05	1.945	0.074	0.00025	1.914	
PJA003	0.01	0.042	1.483	0.217	0.00025	1.567	
PJA004	0.01	0.062	2.219	0.34	0.00025	1.419	
PJA005	0.01	0.056	2.16	0.528	0.00025	1.295	
PJA006	0.01	0.021	1.402	0.304	0.00025	1.145	
PJA007	0.01	0.029	1.665	0.316	0.00025	0.949	
PJA008	0.126	0.035	0.981	0.271	0.00025	1.655	
PJA009	0.021	0.005	0.545	0.096	0.00025	0.772	
PJA010	0.01	0.012	0.767	0.13	0.00025	0.792	
PJA011	0.041	0.014	0.695	0.187	0.00025	0.785	
PJA012	0.023	0.013	0.789	0.173	0.00025	0.74	
PJA013	0.01	0.03	2.402	0.197	0.00025	2.168	
PJA014	0.01	0.005	1.713	0.113	0.00025	1.274	
**Sample ID**	Mg mg/L	Mn mg/L	Na mg/L	Si mg/L	Sr mg/L	Zn mg/L	
PJA001	0.674	0.005	2.078	7.266	0.025	0.018	
PJA002	1.307	0.024	2.927	11.102	0.03	0.011	
PJA003	0.956	0.012	2.296	7.473	0.031	0.005	
PJA004	1.144	0.02	3.272	11.442	0.048	0.005	
PJA005	1.338	0.03	2.587	8.996	0.039	0.005	
PJA006	0.793	0.011	1.587	6.075	0.025	0.005	
PJA007	0.824	0.019	1.556	6.015	0.026	0.005	
PJA008	0.822	0.015	1.245	4.509	0.021	0.005	
PJA009	0.283	0.011	1.002	3.859	0.013	0.005	
PJA010	0.642	0.005	1.012	3.256	0.013	0.005	
PJA011	0.557	0.005	1.111	3.801	0.014	0.005	
PJA012	0.663	0.005	1.219	3.782	0.016	0.005	
PJA013	0.851	0.012	3.792	11.748	0.028	0.005	
PJA014	0.639	0.005	2.159	7.772	0.015	0.005	

**Table 3:** Statistical summary for water results (in mg/L).

Statistical Parameters for Water Samples									Legal References Parameters			
	Element	Detection Limit	Measures > Limit	Mean	Median	Maximum	Minimum	Std. Dv.	PORT.MS 2914/2011	Conama 357	Conama 396	WHO 2011
**Cations**	Al (mg/L)	0.005	4	0.022	0.0100	0.1260	0.0100	0.0312	0.2	0.1	0.2	
	As (mg/L)	0.0001	0						0.01	0.01	0.01	0.01
	B (mg/L)	0.05	0						-	0.5	0.5	2.4
	Ba (mg/L)	0.0005	14	0.029	0.0295	0.0620	0.0050	0.0185	0.7	0.7	0.7	0.7
	Be (mg/L)	0.0002	0						-	0.04	0.004	-
	Ca (mg/L)	0.05	14	1.45	1.50	2.4020	0.55	0.61	-	-	-	-
	Cd (mg/L)	0.0005	0						0.005	0.001	0.005	0.003
	Co (mg/L)	0.0005	0						0.05	0.05	0.05	0.05
	Cr (mg/L)	0.0005	0						-	0.05	-	-
	Cu (mg/L)	0.005	0						2	0.009	2	2
	Fe (mg/L)	0.005	14	0.223	0.1920	0.5280	0.0740	0.1209.	0.3.	0.3	0.3	-
	Hg (mg/L)	0.00009	0						-	-	-	-
	K (mg/L)	0.01	14	1.298	1.2845	2.1680	0.7400	0.4610	-	-	-	-
	Li (mg/L)	0.001	0						-	2.5	-	-
	Mg (mg/L)	0.05	14	0.821	0.8075	1.3380	0.2830	0.2907	-	-	-	-
	Mn (mg/L)	0.001	14	0.013	0.0115	0.0300	0.0050	0.0080	0.1	0.1	0.1	-
	Mo (mg/L)	0.0005	0						-	-	0.07	-
	Na (mg/L)	0.05	14	1.99	1.83	3.7920	1.00	0.90	200	-	200	50
	Ni (mg/L)	0.001	0						0.07	0.025	0.02	0.07
	Pb (mg/L)	0.0005	0						0.01	0.01	0.01	0.01
	Sb (mg/L)	0.0005	0						0.005	0.005	0.005	0.02
	Se (mg/L)	0.005	0						0.01	0.01	0.01	0.04
	Si (mg/L)	0.5	14	6.935	6.6705	11.7480	3.2560	2.9926	-	-	-	-
	Sn (mg/L)	0.001	0						-	-	-	-
	Sr (mg/L)	0.001	14	0.025	0.0250	0.0480	0.0130	0.0104	-	-	-	-
	Ti (mg/L)	0.005	0						-	-	-	-
	V (mg/L)	0.0005	0						-	0.1	0.05	-
	Zn (mg/L)	0.05	2	0.006	0.0050	0.0180	0.0050	0.0037	5	0.18	5	-
**Anions**	Bromide (mg/L)	0.3	1			0.0151			-	-	-	-
	Chloride (mg/L)	1	14	0.89	0.67	3.93	0.48	0.88	250	250	250	
	Fluoride (mg/L)	0.3	14	0.051	0.0442	0.0779	0.0355	0.0141	1.5	1.4	1.5	1.5
	Phosphate (mg/L)	0.5	1			0.0128			-	-	-	-
	Nitrate (mg/L)	0.22	14	0.648	0.6368	1.0463	0.3616	0.1982	10	10	10	50
	Nitrite (mg/L)	0.16	1			0.0101			1	1	1	3
	Sulfate (mg/L)	1	14	0.164	0.185	0.414	0.005	0.109	250	250	250	-
**Physic-Chem Parameters**	Conductivity (µS/cm)	-	14	62.71	56.50	118.00	46.00	19.69	-	100	-	-
	DO (mg/L)	-	14	1.728	1.67	2.67	1.21	0.44	-	>2	-	-
	Temp. (°C)	-	13	25.806	26.20	27.50	24.00	1.16	-	-	-	-
	pH	-	14	6.467	6.44	7.06	6.05	0.29	-	6 to 9	-	-
	Turbidity (NTU)	-	14	289.864	31.90	2016.00	6.50	582.14	-	-	-	-
	Eh (mV)	-	6	−15.786	−2.00	58.70	−149.70	63.96	-	-	-	-
	TDS (mg/L)	-	14	39.929	36.50	78.00	27.00	13.33	-	-	-	-

**Table 4:** Statistical description for stream sediment samples of the study area.

Statistical Parameters for Stream Sediments Samples								Legal References Parameters	
Element	Detection Limit	Measures > Limit	Mean	Median	Maximum	Minimum	Std. Dv	Conama 454 (Level 1)	NOAA SQuiRT 2008 (TEL)
Ag (mg/kg)	0.01	0						-	-
Al (%)	0.01	14	0.127	0.099	0.307	0.033	0.088	-	-
As (mg/kg)	1	10	0.076	0.075	0.240	<LOD	0.072	5.9	5.9
Au (μg/kg)	0.1	14	5.979	5.750	7.000	5.200	0.596	-	-
B (mg/kg)	10	0						-	-
Ba (mg/kg)	5	14	22.833	16.960	53.430	4.090	16.745	-	-
Be (mg/kg)	0.1	8	0.121	0.055	0.670	<LOD	0.182	-	-
Bi (mg/kg)	0.02	0						-	-
Ca (%)	0.01	13	0.013	0.008	0.033	<LOD	0.012	-	-
Cd (mg/kg)	0.05	1			0.100			0.6	0.596
Ce (mg/kg)	0.05	11	7.664	6.435	19.430	<LOD	6.919	-	-
Co (mg/kg)	0.1	14	2.116	1.515	6.140	0.390	1.657	-	-
Cr (mg/kg)	1	14	5.059	3.980	10.700	0.960	3.375	37.3	37.3
Cs (mg/kg)	0.05	9	0.203	0.095	0.530	<LOD	0.212	-	-
Cu (mg/kg)	0.5	11	1.736	1.400	3.900	<LOD	1.359	35.7	35.7
Fe (%)	0.01	14	0.314	0.235	0.718	0.069	0.218	-	-
Ga (mg/kg)	0.1	5	0.264	<LOD	1.600	<LOD	0.521	-	-
Ge (mg/kg)	0.1	14	8.357	7.400	12.800	5.900	2.370	-	-
Hf (mg/kg)	0.05	0							
Hg (mg/kg)	0.01	7	0.132	0.011	1.386	<LOD	0.372	0.17	0.174
In (mg/kg)	0.02	0						-	-
K (%)	0.01	14	0.034	0.024	0.087	0.001	0.028	-	-
La (mg/kg)	0.1	8	1.907	1.250	7.000	<LOD	2.347	-	-
Li (mg/kg)	1	14	1.200	1.000	2.700	0.200	0.760	-	-
Mg (%)	0.01	14	0.040	0.028	0.097	0.003	0.032	-	-
Mn (mg/kg)	5	14	56.579	39.950	170.300	16.200	44.401	-	-
Mo (mg/kg)	0.05	1			0.025			-	-
Na (%)	0.01	1			0.017			-	-
Nb (mg/kg)	0.05	0						-	-
Ni (mg/kg)	0.5	14	1.607	1.400	3.300	0.200	1.058	18	18
P (mg/kg)	50	12	34.857	29.500	8 < LOD	1 < LOD	21.707	-	-
Pb (mg/kg)	0.2	14	2.028	1.845	4.610	0.490	1.382	35	35
Pd (mg/kg)	0.2	0						-	-
Pt (mg/kg)	0.1	0						-	-
Rb (mg/kg)	0.2	1			1.000			-	-
Re (mg/kg)	0.1	0						-	-
S (%)	0.01	0						-	-
Sb (mg/kg)	0.05	0						-	-
Sc (mg/kg)	0.1	13	1.493	1.200	3.500	<LOD	1.001	-	-
Se (mg/kg)	1	0						-	-
Sn (mg/kg)	0.3	1			0.100			-	-
Sr (mg/kg)	0.5	13	1.457	1.050	3.800	0.200	1.121	-	-
Ta (mg/kg)	0.05	0						-	-
Te (mg/kg)	0.05	0						-	-
Th (mg/kg)	0.1	1			0.500			-	-
Ti (%)	0.01	14	0.008	0.007	0.017	0.001	0.005	-	-
Tl (mg/kg)	0.02	0						-	-
U (mg/kg)	0.05	14	0.278	0.201	0.569	0.051	0.176	-	-
V (mg/kg)	1	14	9.426	7.115	23.270	2.290	6.894	-	-
W (mg/kg)	0.1	1			0.100			-	-
Y (mg/kg)	0.05	9	0.750	0.500	2.920	<LOD	0.913	-	-
Zn (mg/kg)	1	7	5.393	4.250	13.000	<LOD	4.896	123	123
Zr (mg/kg)	0.5	0						-	-

**Table 5:** Chemical analysis results of stream sediment samples of the studied area.

Sample ID	Al (%)	As (mg/kg)	Au (mg/kg)	Ba (mg/kg)	Be (mg/kg)	Ca (%)	Cd (mg/kg)	Ce (mg/kg)	Co (mg/kg)	Cr (mg/kg)
PJS001	0.089	<LOD	5.200	11.680	<LOD	0.008	<LOD	2.190	0.780	2.900
PJS002	0.137	0.050	5.500	21.540	0.130	0.005	0.025	8.030	1.550	5.420
PJS003	0.21	0.070	5.700	46.120	0.670	0.031	<LOD	11.090	3.450	9.700
PJS004	0.032	<LOD	5.800	6.340	<LOD	0.001	<LOD	<LOD	0.690	1.390
PJS005	0.171	0.130	7.000	53.430	0.270	0.012	0.100	19.110	6.140	9.070
PJS006	0.249	0.240	6.100	43.580	0.170	0.031	<LOD	19.430	4.040	8.970
PJS007	0.033	<LOD	6.000	4.440	<LOD	<LOD	<LOD	<LOD	0.640	1.560
PJS008	0.099	0.010	5.600	14.190	0.130	0.007	<LOD	2.900	0.800	4.630
PJS009	0.037	<LOD	5.500	4.090	0.030	0.001	0.025	<LOD	0.390	0.960
PJS010	0.196	0.130	5.700	33.240	0.210	0.026	0.025	8.800	3.470	6.590
PJS011	0.045	0.130	7.000	11.660	<LOD	0.033	<LOD	15.390	1.480	2.380
PJS012	0.071	0.080	6.200	12.100	<LOD	0.005	0.025	3.110	2.140	3.230
PJS013	0.307	0.110	5.500	37.520	0.080	0.001	<LOD	12.410	2.640	10.700
PJS014	0.098	0.120	6.900	19.730	<LOD	0.018	<LOD	4.840	1.420	3.330
Sample ID	Cs (mg/kg)	Cu (mg/kg)	Fe (%)	Ga (mg/kg)	Ge (mg/kg)	Hg (mg/kg)	In (mg/kg)	K (%)	La (mg/kg)	Li (mg/kg)
PJS001	0.070	0.800	0.179	<LOD	8.000	<LOD	<LOD	0.027	<LOD	0.600
PJS002	0.210	2.200	0.344	<LOD	6.100	<LOD	<LOD	0.031	1.700	1.100
PJS003	0.520	3.400	0.687	0.300	9.000	<LOD	<LOD	0.086	2.000	2.700
PJS004	<LOD	0.250	0.105	<LOD	7.200	<LOD	<LOD	0.008	<LOD	0.200
PJS005	0.440	3.200	0.485	0.400	12.400	0.022	<LOD	0.045	4.400	1.800
PJS006	0.530	3.500	0.518	1.600	7.400	0.029	0.010	0.065	5.800	1.800
PJS007	<LOD	0.250	0.084	<LOD	5.900	0.021	<LOD	0.004	<LOD	0.500
PJS008	0.110	1.300	0.242	<LOD	11.200	<LOD	<LOD	0.021	<LOD	1.400
PJS009	<LOD	<LOD	0.068	<LOD	6.600	<LOD	<LOD	0.001	<LOD	0.600
PJS010	0.380	2.500	0.407	0.100	7.000	0.344	<LOD	0.066	2.000	2.400
PJS011	0.025	0.800	0.131	<LOD	12.800	1.386	<LOD	0.011	7.000	0.800
PJS012	0.025	0.700	0.191	<LOD	5.900	0.027	<LOD	0.015	<LOD	0.500
PJS013	0.450	3.900	0.7	1.300	7.400	0.022	0.010	0.069	3.000	1.500
PJS014	0.080	1.500	0.22673	<LOD	10.100	<LOD	<LOD	0.020	0.800	0.900
Sample ID	Mg (%)	Mn (mg/kg)	Mo (mg/kg)	Na (%)	Ni (mg/kg)	P (mg/kg)	Pb (mg/kg)	Rb (mg/kg)	Sc (mg/kg)	Sn (mg/kg)
PJS001	0.025	18.600	<LOD	<LOD	0.800	22.000	0.840	<LOD	0.800	<LOD
PJS002	0.038	23.600	<LOD	<LOD	1.500	41.000	2.160	<LOD	1.800	0.050
PJS003	0.097	109.100	<LOD	<LOD	3.300	60.000	3.060	1.000	2.200	0.050
PJS004	0.008	37.900	<LOD	<LOD	0.500	15.000	0.640	<LOD	0.900	<LOD
PJS005	0.046	170.300	<LOD	<LOD	2.600	80.000	4.610	<LOD	2.600	<LOD
PJS006	0.084	86.100	<LOD	<LOD	2.900	66.000	3.550	<LOD	3.000	0.050
PJS007	0.008	18.500	<LOD	<LOD	0.400	10.000	0.490	<LOD	0.800	<LOD
PJS008	0.020	22.900	<LOD	<LOD	1.000	24.000	1.380	<LOD	1.400	<LOD
PJS009	0.003	16.200	<LOD	<LOD	0.200	10.000	0.560	<LOD	<LOD	<LOD
PJS010	0.078	92.800	<LOD	<LOD	2.500	38.000	2.240	<LOD	1.400	<LOD
PJS011	0.016	38.400	0.025	0.017	0.900	46.000	1.850	<LOD	1.000	<LOD
PJS012	0.017	65.100	<LOD	<LOD	1.600	33.000	0.890	<LOD	0.800	<LOD
PJS013	0.082	51.100	<LOD	<LOD	3.000	17.000	4.280	<LOD	3.500	0.100
PJS014	0.031	41.500	<LOD	<LOD	1.300	26.000	1.840	<LOD	0.700	<LOD
Sample ID	Sr (mg/kg)	Th (mg/kg)	Ti_%	U (mg/kg)	V (mg/kg)	W (mg/kg)	Y (mg/kg)	Zn (mg/kg)		
PJS001	0.600	<LOD	0.007	0.177	4.990	<LOD	<LOD	2.500		
PJS002	0.900	<LOD	0.010	0.297	9.990	<LOD	0.660	7.000		
PJS003	3.800	<LOD	0.016	0.460	15.370	<LOD	1.040	1 < LOD		
PJS004	0.400	<LOD	0.002	0.051	2.290	<LOD	<LOD	<LOD		
PJS005	1.900	<LOD	0.009	0.569	17.370	<LOD	2.170	11.000		
PJS006	3.200	<LOD	0.013	0.549	18.550	<LOD	2.920	12.000		
PJS007	0.300	<LOD	0.001	0.073	2.570	<LOD	<LOD	<LOD		
PJS008	1.200	<LOD	0.005	0.191	7.250	<LOD	<LOD	<LOD		
PJS009	0.200	<LOD	0.001	0.178	2.610	<LOD	<LOD	<LOD		
PJS010	2.700	<LOD	0.013	0.340	12.700	<LOD	1.290	13.000		
PJS011	1.700	<LOD	0.002	0.203	3.810	<LOD	0.440	2.500		
PJS012	0.800	<LOD	0.004	0.111	4.210	<LOD	0.090	6.000		
PJS013	0.900	0.500	0.016	0.497	23.270	0.100	1.330	9.000		
PJS014	1.800	<LOD	0.005	0.198	6.980	<LOD	0.560	2.500		

## Data Availability

The geochemical results mentioned in this article can be found in GeoSGB, a database of the Geological Survey of Brazil at http://www.sgb.org.br (accessed on 31 October 2022), or requested from the authors by email.
